# Case Report: Suicidality response to treatment for attention deficit hyperactivity disorder in adult females with autism spectrum disorder: three cases

**DOI:** 10.3389/fpsyt.2026.1767199

**Published:** 2026-04-16

**Authors:** Jessica Hellings, Ishrath Zamani, Megan Evans

**Affiliations:** 1 Department of Psychiatry, University of Missouri-Kansas City, Kansas City, MO, United States; 2 Department of Psychiatry, Medical College of Wisconsin, Milwaukee, WI, United States; 3 University of Missouri-Kansas City School of Medicine, Kansas City, MO, United States

**Keywords:** ADHD, autism, females, suicidality, suicide

## Abstract

**Background:**

Suicidality, suicide attempts and non-suicidal self-injury occur more frequently in untreated attention deficit hyperactivity disorder (ADHD), and in females with autism spectrum disorder (ASD), especially in late adolescence and young adulthood. Diagnosis and treatment of the comorbid ADHD may rapidly improve coping skills, reducing impulsivity and suicidality.

**Methods:**

We obtained IRB approval and written consent to publish the de-identified cases of three young adult females with recurrent suicidality and serious mental illness. Each met DSM-based diagnostic criteria for ASD and ADHD, but received no ADHD treatments on presentation. Presentations, treatment, side effects and precautions are discussed.

**Results:**

Each responded remarkably to ADHD treatments, but with notable side effects especially in one case. Addition of ADHD medications led to rapid improvements in mood, suicidality and self-reported use of coping skills, enabling taper of antidepressants and antipsychotics.

**Conclusions:**

ADHD diagnosis and treatment may rapidly improve treatment-resistant suicidality and mood, by improving executive functions, impulse control and use of coping skills; larger-scale studies are indicated to elaborate on our findings in these three cases. ASD and comorbid ADHD are important predisposing factors to suicidality that are commonly missed. ADHD treatment may provide remarkable response, described by patients as enabling greater functioning, confidence and use of coping skills when under stress. Suicidality assessment should include screenings for ADHD and ASD, especially in atypical cases. Prior maltreatment, executive dysfunction and impulsivity in females all raise suicide risks.

## 1 Introduction

Autism spectrum disorder (ASD) and attention deficit hyperactivity disorder (ADHD) are often overlooked in females with suicidality and serious mental illness, yet they co-occur in 40-70% of ASD cases (1), although studies are not specifically available for females for reasons we will discuss. Suicidality, suicide attempts and non-suicidal self-harm (NSSH) contribute to frequent emergency department (ED) visits, psychiatric admissions and deaths, in these females. A recent study using self-rated screening questionnaires in young adults aged 18–24 years presenting to a generalized outpatient mental health clinic, found 13.13% met the ASD diagnosis (2).

ASD diagnostic criteria include social communication deficits, repetitive behaviors, and restricted interests (3). According to the U.S. Centers for Disease Control (CDC) in 2025, approximately 1 in 31 or 3.2% of children aged 8 years in the U.S. were identified as having ASD in 2022 (4). Another recent study reviewed the lived experiences of those receiving an ASD diagnosis during adulthood, describing their constant struggles and inadequate supports (5). Females are diagnosed later than males, and often have fluent speech, normal IQ, and fewer repetitive behaviors. They may mask or camouflage their differences (6, 7). Therefore the prevalence rate of ASD in women remains unclear (8). ASD gender ratio was originally 4:1 male-to-female, with diagnostic criteria heavily based on male presentations. Recent estimates are 3:1 or lower (9). Many higher functioning females remain undiagnosed (10). ASD females have more depression, anxiety, and ADHD. While some studies found they complete suicide more commonly than males with ASD (11), other studies have found the rates to be more alike and recommend further clarification of this (12).

Self-harm, suicide attempts and suicide are significantly higher in ASD than in the general population. More capable individuals with ASD have greater suicide risks and are more likely to die of suicide than their lower-functioning counterparts, who are more likely to die from physical causes (13). Women in the general population without ASD, in 2022 had approximately 6 deaths by suicide per 100,000, according to the U.S. National Center for Health Statistics (NCHS) (14). Women with ASD are 5–13 times more likely to complete suicide than those without ASD (15). The same study found that delayed diagnosis increases suicide risk, as do impulsivity, cognitive inflexibility, social isolation, adverse life events and camouflaging.

Suicide is a leading cause of death in females with ASD; two-thirds consider suicide during their lives (16). Over half later plan or attempt suicide. This is versus 18% of females considering suicide in their lifetime in the normal population, based on Australian Institute of Health and Welfare 2020–2022 data (17). While completed suicide is more common in men in general, high-functioning females with ASD may have higher risk than male counterparts. The term “high functioning” is not formally defined (18) however it refers to individuals with average or above average intelligence, including language and basic abilities, accompanied by social communication and executive function impairments, rigidity and sensory sensitivities. In the Diagnostic and Statistical Manual-5 Text Revised (3), these individuals would meet Level 1 ASD criteria. Suicidality often occurs in ASD females without clinical depression.

ASD is often associated with ADHD, in 50-70% (1) however the ADHD frequently goes untreated. Although ADHD diagnostic criteria include impaired focus, concentration, and impulse control, associated executive function (EF) deficits produce problems in education, work, relationships and finances. EF manage working memory, planning and adaptive activities and impulsivity inhibition. In ASD, EF deficits are more generalized and more severe than in ADHD alone (19). Impulsivity worsens behavioral outbursts, aggression, and suicidal behaviors, especially with prior maltreatment or bullying (20). Instead of ADHD, such problems may be attributed to comorbid PTSD or other disorders.

We describe 3 cases illustrating severity of psychopathology. All had suicidality on antidepressants and antipsychotics. We obtained IRB permission from University of Missouri-Kansas City, written consent from two patients, and for one, guardian consent and patient assent. We use the CARE (CAse REport) guidelines (21) to describe 3 young adult females presenting with suicidality. Case dates/timeline were however removed per IRB request.

## 2 Case descriptions

All three adult females met ASD and ADHD criteria in DSM-5 (22) for those diagnosed before 2022, and DSM-5-TR (3) criteria for one diagnosed later. Each rated strongly positive on the Adult ADHD Self-Report Rating Scale (23) for ADHD, and did not respond to antidepressants and antipsychotics. Final Clinical Global Impressions-Improvement subscale ratings (24) in all cases was Much Improved following ADHD treatment. ED visits and suicidality decreased dramatically. See **Tables 1**, **2** for case summaries.

**Table 1:** Past history and symptoms of each female.

Case	Age	Race and/or ethnicity	Gender	Early childhood history	ASD symptoms	ADHD symptoms	Maltreatment	Suicidality	Suicide attempts	Non-suicidal self-injury
Case 1	21	White	Female	Talked late at 5 years, ADHD diagnosed at age 9 years	Talks mainly about restricted interests, hard to converse, socially isolated, poor social judgment, Problems with noises and textures, likes soft toys, play-do as an adult, hoarding	Hard to stratify her needs and focus on them, inattentive, fidgety, restless, impulsive sexual risks, explosive, property destruction, self-harm	bullied in school, abused by men on internet, boyfriends raped her	Age 16 admission for SI, 2 admissions for SI aged 18, and for suicide plan aged 20	8 attempts from age 16 onwards, age 20 by waterboarding, also hanging	Scratched arm, head-banged to concussion
Case 2	24	White	Female	Mild ID and ASD diagnosed at age 7, never got along with mother	Liked watching violent movies, socially inappropriate	Short focus, needs reminders, distracted by noises	Interactional problems with mother, sexual abuse by father at age 13.	Repeated psychiatric admissions after age 12	at age 21 hospital visits x 6 for wrapping phone cord around neck	Scratched self with nails, leaving marks
Case 3	18	White	Female	Interactional difficulties, home schooled, temper outbursts	Watched same shows over and over, different from peers, hard to converse or make friends, misophonia	Poor focus, disorganized, unable to plan and achieve goals, impulsive, risk-taking behaviors, forgetful	Bullied in school, suspended for punching a boy, and a girl (separate incidents)	Age 12 started sertraline, activated her, ED visit for SI. Frequently suicidal but without plans	Drug overdose at age 14	Non-suicidal self-cutting 10 times, ages 14-16

ADHD, attention deficit hyperactivity disorder; ED, emergency department; SI: suicidal ideation.

**Table 2:** Treatment history and outcomes timeline for each female.

Case	Age	Race and/or ethnicity	Gender	Date and medications on presentation	Diagnoses before presentation	Final diagnoses	Date and final medications	Individual therapy	Final Outcomes
Case 1	21	White	Female	12 September, 2022 lurasidone 20mg bedtime, mirtazepine 7.5mg bedtime, sertraline75mg daily	Major depression, PTSD, GAD, Borderline personality disorder, ADHD	Triple X chromosome ASD, ADHD, PTSD, GAD, phobia of textures	28 January, 2025 Mixed amphetamine salts 15mg am and 3pm, Mirena IUD [TM] adalimumab i.m.i. weekly for hydradenitis, tetracycline for skin abscesses	Yes, suicide-focused, helped but stopped due to 3 missed appts, had been much improved	CGI-S= 4 (Moderate problem) CGI-I= 1 (Very much improved)
Case 2	24	White	Female	30 April, 2019 Olanzapine 5mg am, Paliperidone 6mg qhs, sertraline 200mg daily, doxepin 10mg bedtime prazosin 2mg bedtime lamotrigine 100 twice/day, melatonin 3 mg bedtime trazodone 100mg bedtime, hydroxyzine 50 mg PRN q6hrs, l-thyroxin 0.025mg daily, loratidine 10mg daily, doxycycline 100 twice daily for acne, multivitamin daily	Major depression, PTSD	ASD, ID- mild, ADHD, PTSD, IED	10 April, 2025 atomoxetine 40am, 25 mg bedtime, lamotrigine 100mg am, 75 mg hs, prazosin 6mg hs, melatonin 9mg hs, zolpidem 5mg hs PRN, l-thyroxin 0.025 mg daily, doxycycline 100 mg twice/day (acne), loratadine 10 mg daily, Nexplanon birth control subcutaneous, Vit D3 2000 iu daily	Not established yet	CGI-S= 3 (Mild problem) CGI-I= 1 (Very much improved)
Case 3	18	White	Female	12 February, 2019 bupropion XL 300mg dly, aripiprazole 10 mg dly, melatonin 10 mg qhs, hydroxyzine 75mg PRN daily for anxiety, cannabidiol oil 10 mg daily medroxyprogesterone i.m.i. every 3 months (birth control)	Major depression, bipolar disorder, GAD misophonia	ASD, ADHD-combined, GAD, recurrent adjustment disorder with depressed mood, and misophonia	7 November, 2024 Mixed amphetamine salts ER 25mg qam, mirtazapine 15 mg bedtime, Birth control pill daily, propranolol 10mg qam plus 10mg extra as needed for palpitations	Ongoing, helpful	CGI-S= 3 (Mild problem) CGI-I= 2 (Much improved)

ASD, autism spectrum disorder; CGI-S, Clinical Global Impressions scale-Severity.

ADHD, attention deficit hyperactivity disorder; CGI-I, Clinical Global Impressions scale-Improvement.

GAD, generalized anxiety disorder; am, in the morning.

ID, intellectual disability; hs, at bedtime.

IED, intermittent explosive disorder; appts, appointments.

PTSD, post-traumatic stress disorder.

### 2.1 Case 1

21-year-old Caucasian female, previously managed at a university outpatient psychiatry clinic, requesting an autism evaluation. Current psychiatric diagnoses were post-traumatic stress disorder (PTSD), generalized anxiety disorder (GAD), major depression and bipolar disorder.

#### 2.1.1 Main complaint

Her mother described the patient’s difficulties “stratifying her needs and prioritizing issues”. She focused on preferred interests and was inattentive, fidgety and restless, with outbursts of yelling and breaking things impulsively. She was frequently suicidal.

#### 2.1.2 Developmental history

She talked late, at age 5. She was bullied and disciplined in school for socially inappropriate behavior. She conversed about preferred topics rather than reciprocally, and hoarded objects. She stayed home alone rather than socializing. She described difficulty tolerating textures. Eating noises of others upset her (misophonia). Clinically, she met ASD diagnostic criteria.

#### 2.1.3 Past psychiatric history

Childhood ADHD was diagnosed and several medication trials were done. Details of the latter are unknown however she recalled one medication had kept her awake all night (likely a stimulant since insomnia is a common side effect; she related an inability to tolerate several medications tried due to side effects). She described internet harassment as a teenager by older men, nightmares and flashbacks of abuse involving boyfriends, and admission at age 16 for SI. At age 18, she was diagnosed with major depression twice during inpatient admissions for suicidality. She denied using cigarettes or alcohol and tried cannabis infrequently.

From age 16 she attempted suicide over 5 times, by cutting, strangulation, suffocation, head-banging and waterboarding. She was admitted for planning to jump off a bridge; inpatient psychiatry notes documented her mood as euthymic.

#### 2.1.4 Medical history

Medical history included Triple X syndrome [age at diagnosis unclear, and is associated with ADHD, psychosis and learning disorders (25)], asthma, seasonal allergies, hydradenitis, skin abscesses, and constipation. She consulted gastroenterology for food allergies and abdominal pains since her youth. Risperidone treatment (exact indication unknown though indicated for treatment of irritability) previously caused excessive weight gain.

#### 2.1.5 Mental status examination

She was reasonably kempt, fidgety and restless with little eye contact. Expressive language was adequate. She described her mood as neutral. She denied current suicidal ideation; reporting 2 weeks since last SI. She denied hallucinations or delusions. Memory was intact, orientation normal. Judgment and insight were poor, based on risk-taking and impulsive behavior.

#### 2.1.6 Medications on presentation

Medications on presentation were lurasidone 20mg bedtime (indication unclear but likely an add-on for treatment-resistant depression), mirtazapine 7.5 mg bedtime, and sertraline 75 mg daily. She attended weekly counseling targeting suicide prevention.

#### 2.1.7 Psychiatric diagnoses

Psychiatric diagnoses made at this initial evaluation were ASD, ADHD-combined type, PTSD, GAD, and major depression by history.

#### 2.1.8 Follow-up

At first follow-up she had discontinued all the above medications due to lack of efficacy. The Adult ADHD Self-Report scale was strongly positive. She was prescribed mixed amphetamine salts (Adderall TM) 10mg morning and 2pm after discussion of effects and side effects.

At second follow-up she described being “able to do things” including chores; difficulties recurred if medication was forgotten. She denied SI since her previous visit. At third follow-up, she again denied any SI and reported doing well. On fourth follow-up she and her mother reported she was much improved, stating this was the first medication that had helped, and improved her use of coping skills. Her stimulant medication was continued the same for two follow-ups. Then at her request, the stimulant dose was increased to 15mg morning and at 3.30pm daily. She continued to function well overall, whereas before this medication, outbursts were at least daily. She worked a part-time job and attended community college.

She continued with neutral mood, while doing well, confirmed by mother. She denied SI, psychiatric admissions or ED visits for 25 months.

#### 2.1.9 Side effects

These include an episode of palpitations (heart rate 97 bpm); she refused metoprolol 25mg twice daily from cardiology, after serious causes were excluded. Her weight had always fluctuated; BMI during treatment varied between 17.5-19.

#### 2.1.10 Final psychotropic medications

Final psychotropic medications were mixed amphetamine salts 15mg morning and 3pm daily.

#### 2.1.11 Final psychiatric diagnoses

Final psychiatric diagnoses were ASD, ADHD-combined, PTSD and GAD. In terms of high-normal cognitive functioning, she was able to complete an associates degree at a local community college while receiving the ADHD treatment described above.

### 2.2 Case 2

24-year-old Caucasian female presented with a caregiver, previous treatment site information unknown; and she attended a day program. While details of activities in the day program were not stipulated, these generally involve individual and group activities and outings with peers, along with staff supervision.

#### 2.2.1 Main complaint

The caregiver reported she “needs to focus, and control her behaviors”, and needed constant prompts because of distractibility, causing difficulties following directions. She impulsively threw items including chairs, yelled and cursed, threatening to kill herself and others, and was frequently suicidal. She previously made scratch marks breaking her skin. These symptoms suggested ADHD, apart from her mild ID diagnosis, noting that diagnostic overshadowing is a term describing incorrect attribution of inattention, distractibility and impulsivity of ADHD to the ID.

#### 2.2.2 Past psychiatric history

Previous diagnoses were ASD and mild ID. She received psychotropic medications during childhood, details unknown. She described interactional problems with her parents, abuse by her father, elopement, home escorts by police, and molestation in her early teens. She was later diagnosed with post-traumatic stress disorder (PTSD), with nightmares, flashbacks, and traumatic visualizations. She had repeated psychiatric admissions as a preteen. In her early twenties she had over 5 ED visits for attempted self-strangulation, and recent ED assessment for threats of hurting herself and others.

#### 2.2.3 Mental status examination

She was well kempt, distractible, with adequate language, and mood “so-so”. She denied suicidal or homicidal ideation, hallucinations or delusions. Orientation and memory were normal, judgment and insight poor, and fund of knowledge below average.

#### 2.2.4 Medical history

Medical history included obesity, acne, migraines, hypothyroidism, irregular menses, and urinary tract infections. She denied substance use.

#### 2.2.5 Psychotropic medications on presentation

Psychotropic medications on presentation were olanzapine 5mg morning, paliperidone 6mg bedtime, sertraline 200mg daily, doxepin 10 mg bedtime, prazosin 2mg bedtime for nightmares, lamotrigine 100mg twice daily for mood stabilization, melatonin 3 mg bedtime, hydroxyzine 50mg PRN 6 hourly for anxiety, and trazodone 100mg bedtime PRN.

#### 2.2.6 Diagnoses made at presentation

Diagnoses made at presentation included ASD, ADHD combined type, intermittent explosive disorder, PTSD and mild ID.

#### 2.2.7 Follow-up and treatment

Recommended medication changes included reducing sertraline gradually to 100mg daily, due to possible activation and SI worsening by the high dose. Olanzapine was reduced to 2.5mg daily for 3 days then discontinued. Atomoxetine was added for ADHD, notably 18mg at bedtime, then 5 days later increased to 18 mg twice daily. Loxapine 5 mg at bedtime was added for irritability, since clinical experience experience suggests more weight neutrality, and paliperidone reduced to 3 mg at bedtime then later discontinued.

#### 2.2.8 Follow-up

On first follow-up she reported another psychiatric admission, following elopement and police intervention. Since starting atomoxetine however she was less explosive, and calmed sooner if frustrated. Her caregiver confirmed this. Atomoxetine was increased to 25 mg twice daily. Six weeks later, outbursts had stopped, and she reported feeling good overall. Almost 3 months later, her behavior remained much improved. Her mood was good, with occasional flashbacks. She denied suicidal or homicidal ideation.

Follow-up visits continued every 1–2 months. During covid-19 pandemic accompanied by isolation, she experienced more mood lability, and scratched her skin. Sertraline was reduced then stopped, due to likely activation effects, this was tolerated well. Atomoxetine for ADHD was increased to 40mg morning, 25mg bedtime, producing improvement which continued for almost 6 years. She still attended a day program. Despite several clinician efforts to establish trauma-focused psychotherapy for her, it is notable that this was never accomplished.

#### 2.2.9 Final psychiatric medications

Final psychiatric medications (See **Table 2**) were atomoxetine 40mg morning, 25mg bedtime for ADHD, lamotrigine 100mg twice daily (mood stabilization), prazosin 6mg bedtime, melatonin 9mg bedtime, zolpidem 5 mg bedtime PRN (used occasionally), hydroxyzine 25 mg at 4pm daily for anxiety.

### 2.3 Case 3

18-year-old Caucasian female assessed in high school final year, asking for autism assessment “since she was different from her peers”. Previous treating clinician affiliation is unknown. She was accompanied by her father to several of the early visits.

#### 2.3.1 Main complaint

While her school grades were good in a mainstream class in high school, daily interactional challenges including peer bullying and her inability to understand jokes produced suicidality. She described noise sensitivity in class, and misophonia. Boys made inappropriate sexual comments, and girls antagonized her. She had school suspensions, cut her skin superficially, and felt she was a failure. She walked outside late at night, needing police escorts home.

#### 2.3.2 Past psychiatric history

She took sertraline (dose unknown) as a pre-teen, resulting in activation, after which aripiprazole 5 mg twice daily was added, along with a diagnosis of bipolar mood disorder. She underwent ED evaluation after impulsively overdosing on <10 naproxen tablets, following peer bullying; she did not want to die. Following high school, she dropped out of community college; work was also challenging. She often skipped her medications, due to tiredness and inefficacy.

#### 2.3.3 Psychotropic medications on presentation

Psychotropic medications on presentation (See **Table 2**) were bupropion XL 300mg daily, aripiprazole 10mg daily, melatonin 10mg bedtime, hydroxyzine hydrochloride 75 mg PRN daily for anxiety, and up to 10mg cannabidiol oil daily. She denied substance abuse. She received individual psychotherapy weekly or semi-weekly.

#### 2.3.4 Family history

Two relatives took mixed amphetamine salts for ADHD.

#### 2.3.5 Developmental history

Developmental history was without obvious delays, but with more outbursts than her siblings. She watched the same movies repetitively, and was home-schooled initially due to interactional problems.

#### 2.3.6 Medical history

Previous anemia.

#### 2.3.7 Mental status examination

She appeared well-groomed and euthymic, denied suicidal or homicidal ideation, hallucinations or delusions. Memory was intact with poor judgement and insight.

#### 2.3.8 Diagnoses

Diagnoses were ‘rule-out’ autism spectrum disorder at this visit, and bipolar disorder by history. Medications were initially continued the same.

#### 2.3.9 Follow-up and treatment

In first follow-up visit, she reported experiencing intermittent SI, without plans. She complained of long-standing poor focus and disorganization, also remarked on by others, suggesting ADHD.

Atomoxetine 10mg twice daily was begun, and bupropion XL tapered gradually to 150mg daily. She was alerted that bupropion interacts with atomoxetine by increasing its effects. Mood and functioning improved, with reduced SI.

Next visit she complained of some mood swings, while still taking on bupropion XL 300mg daily; reduction to 150mg daily reduced these. Aripiprazole was tapered off gradually without problems. Related to family members’ response to mixed amphetamine salts i.r for ADHD, this stimulant was added at her request, at 7.5 mg morning and midday, 2.5mg at 5pm, again producing improvement. The dose was increased at her request to 10mg morning and 2pm, and 2.5mg at 5pm, along with atomoxetine 10 mg bid, and bupropion XL 150mg daily.

She later became suicidal again under stress for a few days, without plans. She requested another dose increase of stimulant, to 15 mg morning and lunch, 2.5mg evening. Thereafter she reported improved functioning and mood without further suicidality.

She became more stable overall. She requested stimulant change to the ER form, notably 15mg ER morning and 3pm. She later, without consultation combined both doses (30mg) in the morning. She tolerated taper off bupropion. Shortly after atomoxetine was increased to 18 mg twice daily, together with the large morning dose of ER stimulant, she experienced a supraventricular tachycardia (SVT). She reported drinking energy drinks and excessive coffee daily prior to the arrhythmia, but reported she stopped this habit, afterwards and going forward. Psychotropic medications were stopped in the ED. While off psychotropic medications, a lack of concentration recurred, with hopelessness but no suicidality. Following a week of Holter monitoring cardiology approved restarting the stimulant in ER form, 25mg daily, but not its combination with atomoxetine. Her general practitioner (GP) prescribed propranolol 20 mg daily, and 10 mg extra PRN daily for palpitations. She reported being compliant with her medications. Her GP added mirtazepine 15mg bedtime for insomnia with good results.

She denied SI for >1 year. She worked part-time, and socialized more. She used recreational cannabis 5 mg chews once or less per week. Total treatment duration was 5 years, 9 months.

#### 2.3.10 Final psychiatric diagnoses

Final psychiatric diagnoses were ASD, ADHD-combined, recurrent adjustment disorder with depression and SI in remission. The bipolar disorder diagnosis was removed since activation and rapid thoughts had occurred with antidepressants. While formal testing was not available to validate her high-functioning status, she had good grades in her mainstream classes in high school, and she was given a scholarship to a local community college. When her ADHD was treated she was able to hold a part-time job for several years and train other staff in it. She reported writing plays in her spare time.

#### 2.3.11 Final psychotropic medications

Final psychotropic medications were mixed amphetamine salts ER 25 mg morning, propranolol 10mg morning plus extra 10 mg PRN daily (palpitations), and mirtazapine 15 mg nightly.

#### 2.3.12 Side effects

The SVT described above did not recur. Her BMI increased from 27.2 to 35.2. She refused weight-related interventions, including stopping mirtazapine or cannabis, both of which produce weight gain.

## 3 Discussion

The cases highlight delays in obtaining ASD and ADHD diagnosis and treatment, also suicidality, suicide attempts, and serious mental illness (See **Tables 1**, **2**). Patients’ adverse experiences, abuse, and severity of psychopathology together with rapidity of suicidality response to ADHD medications are remarkable, along with self-reported increased ability to use coping skills. Mood improved despite antidepressant tapers; antidepressant-induced worsening and behavioral activation in ASD is a significant side effect (26). In adolescents and young adults, antidepressants differ only slightly from placebo in terms of their antidepressant effects, according to a 2021 Cochrane review (27). Also in youth, antidepressants reduce suicidal tendencies less than they induce them.

The package inserts of all antidepressants include a boxed warning (the strongest possible warning) for suicidal thoughts and behavior, in children and young adults, which are highest when they are first started and may occur with dosage changes.

Antipsychotics can increase suicidality if they cause motor restlessness (akathisia), though more data on this is needed (28). While Cases 1. and 2. received medication treatments during childhood, exact details are unknown, and it is likely that various medications could have been stopped due to side effects. The medication that kept Case 1. awake all night as a child was likely a stimulant, and individuals with ASD are more prone to stimulant side effects (29), as discussed more below. More longitudinal studies of medications in ASD are needed.

Such females as described here in Cases 1 and 3 often appear bright, have good language skills, may make eye contact and are able to function partially in school and work settings. Positive behaviors may mask their difficulties of rigid thinking, social isolation, inattention, impulsive behaviors and suicidality when under stress. It is also common for ADHD and ASD to be overlooked diagnostically in females and in adults in general, though there is a call for ADHD also to be added to the list of severe mental illnesses. The latter, which would be differential diagnoses for suicidality, include major depression, bipolar disorder, schizophrenia, anxiety disorders, substance use disorders and personality disorders.

Heterogeneity is present among the three cases described. Case 1. made serious suicide plans and attempts and was at extremely high risk for suicide. Case 2. in contrast to the other two females, had mild ID and had experienced more family chaos and abuse by at least one and possibly both parents, and greater deprivation. She did not live independently but with a paid caregiver, attended a day program and had a case manager. In spite of severe PTSD even with clinician help she did not establish any kind of trauma-focused psychotherapy. Each of the other two females had one or both parents accompany them to clinic visits, whereas Case 2. did not have such family supports. She did not have the capacity as an adult to work in the open job market. In addition, Case 1. was maintained on stimulant monotherapy and was underweight, while Case 3. was treated with a stimulant along with propranolol, and mirtazepine, the latter medication likely contributing to obesity. Case 2. had been more physically aggressive prior to her ADHD treatment, had hospital admissions for suicidality and her final medications at the time of this reporting included several more psychotropics than the ADHD medication atomoxetine. Nevertheless, treatment of their ADHD with medications in all cases, provided rapid improvements in suicidality, and self-reported greater ability to use coping skills under stress.

ADHD medications including stimulants however produce more side effects in individuals with ASD (29). Case 1. responded to a stimulant alone. Case 3. had an SVT on the long-acting stimulant in relatively high dose together with atomoxetine and caffeinated drinks. Case 2. responded well following atomoxetine addition. While atomoxetine has a black box warning for SI, a large study comparing it to stimulants found no higher risk of suicidal events in youths treated with atomoxetine (30). Cardiovascular risks of stimulants are higher also in individuals with obesity. Obese individuals are more likely to develop hypertension and cardiovascular disorders, which are also risks of chronic stimulant use (31). Treatments for ADHD other than medications, also advisable in combination with ADHD medications, include cognitive behavioral therapy, outpatient psychotherapy for those with high-functioning ASD (32), structured environments, executive function coaching, exercise, dietary interventions and supplements (33).

A study of largely female individuals who had attempted suicide more than once found >40% had significant ASD traits (34). Another study (35) assessed self-report standardized rating scales in 63 adult psychiatric outpatients with ASD (57% females) to assess SI, NSSI and suicide attempts. Almost one third (31%) experienced SI in the past month, with lifetime SI in 86%. One quarter had made at least one suicide attempt. Substance use and depression were risk factors. Almost half (44%) performed NSSI; more commonly women. Substance use was commonly overlooked, particularly in women. Another study (36) found exacerbating factors for suicidality in individuals with ASD without ID were cognitive rigidity, rumination, and emotional regulation difficulties. Comorbid conditions including ADHD, depression, and addiction were other risk factors.

Risks for suicidality in one population-based study (37) were highest in males and females with ASD without ID if they had comorbid ADHD. They also made more severe suicide attempts requiring inpatient treatment, and suicides. Another study (20) explored maltreatment effects on outcomes in 140 girls and women with ADHD but not ASD evaluated in childhood, adolescence and young adulthood. Physical or sexual abuse or neglect occurred in 22.9%. Maltreated females had more internalizing behaviors, including suicide attempts, self-harm, anxiety, depression, and eating disorders. A study (38) investigated whether ASD could underlie both suicidality and borderline personality disorder characteristics. They found 10% overlap between the two diagnoses, advising ASD screening in atypical presentations accompanied by persistent suicidality.

### 3.1 Study limitations

Improvements cannot be attributed to ADHD medications alone, since other psychotropics were taken in two cases. Related to polypharmacy, the extent to which drug interactions and adverse effects influenced the symptoms in these cases cannot be determined retrospectively. Side effects and interactions of each of the medications are listed in their package inserts. Also, stimulant medication may help improve attention and thus overall status in other psychiatric disorders apart from ADHD (39), although a common side effect clearly stated in stimulant product information is sleep disorders including insomnia (40). In addition, tapering cautiously off antidepressants which may have been worsening suicidality along with activation and irritability could have contributed to improvements seen, although it is not possible to verify the extent to which taper off medication alone contributed to decreases in suicidality. Also, prazosin could contribute to improvements by improving sleep, and reducing nightmares. Likewise propranolol, by reducing anxiety could improve overall status. Environmental factors including social supports could also have contributed to improvements. Another weakness is that none of the cases had a comprehensive treatment plan, and Case 2. despite having a case manager lacked any trauma-focused therapy in spite of her good verbal skills. The ADHD Self-Report Rating scale can strongly suggest ADHD but clinical diagnosis is needed. Nevertheless, the improvements in each case after ADHD medication addition support ADHD diagnosis, though they cannot prove it. Likewise the ASD diagnoses were made clinically using DSM criteria. Larger-scale studies are needed to confirm our findings.

## 4 Conclusion and primary take-aways

All three females described with ASD presented with suicidality and non-suicidal self-injury. Our review and case descriptions illustrate the importance of screening for multiple suicidality risk factors, especially untreated ADHD and undiagnosed ASD, particularly in females presenting with repeated life stresses and failures along with suicidality. Females with ASD and ADHD could benefit from more accurate and earlier diagnosis, and treatment with medication, psychotherapy and appropriate supports. In addition it is important to monitor side effects of medications used, along with intake of caffeine. Larger-scale studies are also needed, that screen females with suicidality, suicide attempts and non-suicidal self-harm for ASD and ADHD.

## 5 Patient perspectives

Case 1: The stimulant was the first medication that had helped her accomplish goals, and improved use of coping skills and reduced suicidality. If she forgot the medication, she noticed more difficulties again.

Case 2: Remarked on improved mood and greater ability to calm down if frustrated, and to use coping skills rather than become suicidal.

Case 3: Reported better mood and accomplishment of goals, using coping skills, unless she forgot her medications or also when they were stopped after an SVT.

## Data Availability

The original contributions presented in the study are included in the article/supplementary material. Further inquiries can be directed to the corresponding author.
